# A comprehensive immune repertoire study for patients with pulmonary tuberculosis

**DOI:** 10.1002/mgg3.792

**Published:** 2019-06-07

**Authors:** Yingyun Fu, Bo Li, Yazhen Li, Minlian Wang, Yongjian Yue, Lan Xu, Shulin Li, Qijun Huang, Song Liu, Yong Dai

**Affiliations:** ^1^ Department of Respiratory and Critical Care Medicine The Second Medical College of Jinan University (Shenzhen People’s Hospital) Shenzhen China; ^2^ Shenzhen Key Laboratory of Respiratory Disease Shenzhen China; ^3^ Department of Pediatrics The Second Medical College of Jinan University (Shenzhen People’s Hospital) Shenzhen China; ^4^ Clinical Medical Research Center The Second Clinical Medical College of Jinan University (Shenzhen People’s Hospital) Shenzhen China

**Keywords:** CDR3 sequences, high express clones, high throughput sequencing, TRBJ gene, tuberculosis

## Abstract

**Background:**

Tuberculosis (TB) is a major global health problem and has replaced HIV as the leading cause of death from a single infectious agent.

**Methods:**

Here, we applied high throughput sequencing to study the immune repertoire of nine pulmonary tuberculosis patients and nine healthy control samples.

**Results:**

Tuberculosis patients and healthy controls displayed significantly different high express clones and distinguishable sharing of CDR3 sequences. The TRBV and TRBJ gene usage showed higher expression clones in patients than in controls and we also found specific high express TRBV and TRBJ gene clones in different groups. In addition, six highly expressed TRBV/TRBJ combinations were detected in the CD4 group, 21 in the CD8 group and 32 in the tissue group.

**Conclusion:**

In conclusion, we studied the patients with tuberculosis as well as healthy control individuals in order to understand the characteristics of immune repertoire. Sharing of CDR3 sequences and differential expression of genes was found among the patients with tuberculosis which could be used for the development of potential vaccine and targets treatment.

## 1 INTRODUCTION

Tuberculosis (TB) is a major global health problem and has replaced HIV as the leading cause of death from a single infectious agent in 2016. In 2015, an estimated 10.4 million people developed TB and 1.4 million died from the disease. Tuberculosis is a disease caused by mycobacterium tuberculosis infection. The immunology reaction caused by pathogens during the infection and reproduction of mycobacterium tuberculosis has been studied which leads to the development of vaccine, diagnosis, drug resistance (Horwitz, Lee, Dillon, & Harth, 1995; Lindenstrøm et al., 2009; Meintjes et al., 2009; Vanham et al., 1997). Since, the TCR repertoire is a mirror of the human immune response, its characteristics have been widely investigated in infectious and other diseases to study the state of the immune system and the progression of these diseases (Chaudhry, Cairo, Venturi, & Pauza, 2013).

The diversity within the TCR repertoire is ensured through somatic recombination of germline‐encoded variable (V), diversity (D), and junctional (J) gene segments. Nucleotide deletions at the coding ends and nucleotide additions at the V(D)J junctions also contribute substantially to the TCR repertoire diversity (Nikolich‐Žugich, Slifka, & Messaoudi, 2004). The TCR diversity is a function of the third hypervariable complementary‐determining (CDR3) region, which lies at the intersection between the V, D, J and V, J gene segments within the TCR and TCR chains, respectively. The CDR3 region encodes that part of the TCR which predominantly interacts with antigenic peptide/MHC complexes. Thus, even when T cell clones express the same V/J genes rearrangement, they can be identified by the unique combination of their CDR3 sequences (TCR clonotypes) (Toivonen, Arstila, & Hänninen, 2015). Accordingly, the complexity and distribution of TCRs within specific T cell populations will reflect the degree of complexity of the T cell response.

In the present study, we studied the immune repertoire of CD4^+^, CD8^+^ T cells of patients and healthy controls and tissue sample of patients to elucidate the effect of tuberculosis on patients’ immune system. The characteristics of diversity and stability, CDR3 length distribution and CDR3 sequences sharing were analyzed. Besides, the usage of TRBJ, TRBV as well as the combination of TRBV/TRBJ were studied. The different repertoire features between tuberculosis patients and controls were then found as future targets for further study.

## 2 METHODS

### 2.1 Clinical samples

Tuberculosis tissue samples and blood samples of nine patients and blood samples of nine healthy controls were collected at the Second Clinical Medical College of Jinan University (Shenzhen People's Hospital, Guangdong, China). All patients gave written informed consent and the present study was approved by the Medical Ethics Committee of Shenzhen People's Hospital.

### 2.2 DNA extraction and mixing

T cell was isolated using superparamagnetic polystyrene beads (Miltenyi) coated with monoclonal antibodies specific for T cells. DNA was prepared from 0.5 to 2 × 10^6^ T cells from each sample (patients and controls), which was sufficient for analyzing the diversity of TCR in the T cell subsets. DNA was extracted from PBMCs using GenFIND DNA (Agencourt, Beckman Coulter, Brea, CA) extraction kits following the manufacturer's instructions.

Ten milligrams of tuberculosis tissue was obtained from each patient sample and DNA was extracted using standard methods. Briefly, dewaxing was done using xylene and followed by over‐night proteinase K digestion for tissues. QIAamp DNA Mini kit (Qiagen GmbH, Hilden, Germany) was further used for DNA extraction following the manufacturer's instructions. DNA quality was evaluated by loading on a 0.8% agarose gel electrophoresis and DNA concentration was quantified by Qubit fluorometer. DNA from nine patients' peripheral blood samples were mixed together by 1:1:1:1:1:1:1:1:1 according to Qubit value, renamed one blood sample. Meanwhile, DNA from nine patients' tuberculosis tissues and control blood samples were mixed separately in the same way.

### 2.3 Multiplex‐PCR amplification of TCR‐β CDR3 regions

The human TCR‐β sequences were downloaded from IMGT (http://www.imgt.org/). A relative conserved region in frame region 3, upstream of CDR3, was selected for the puta‐ tive forward primer region. A cluster of primers corresponding to the majority of the V gene family sequence was selected. Similarly, reverse primers corresponding to the J gene family were designed. In total, 30 forward primers and 13 reverse primers were used for multiplex PCR to amplify the rearranged TCR‐β CDR3 regions. The reaction mixtures (50 μl total) comprised 2 μl of pooled TCR‐β variable gene (TRBV; 10 μM), 2 μl of pooled TCR‐β joining gene (TRBJ; 10 μM), 25 μl of 2X Qiagen Multiplex PCR Master Mix, 5 μl of 5X Q‐solution, 500 ng of template DNA (10 μl) and 6 μl of H_2_O. The PCR conditions comprised 95 °C for 15 min; followed by 25 cycles of 94 °C for 15 s and 60 °C for 3 min; followed by a final extension for 10 min at 72 °C. The PCR products were purified using AMPure XP beads to remove primer sequences (Beckman Coulter, Inc., Brea, CA, USA). A second round of PCR was performed to add a sequencing index to each sample. In this round, each reaction mixture (50 μl total) consisted of 13.5 μl of H_2_O, 0.5 μl of 2X Q5 DNA polymerase, 10 μl of 5X Q5 buffer, 1 μl of dNTPs (10 mM), 1 μl of P1 (10 μM), 23 μl of DNA, and 1 μl of index (10 μM). The PCR conditions comprised 98 °C for 1 min; followed by 25 cycles of 98 °C for 20 s, 65 °C for 30 s and 72 °C for 30 s; and a final extension for 5 min at 72 °C. The library was separated on an agarose gel, and the target region was isolated and cleaned using QIAquick Gel Extraction kits (Qiagen).

### 2.4 NGS and data analysis

The library was quantitated using the Agilent 2100 Bioanalyzer instrument (Agilent DNA 1,000 reagents) and real‐time quantitative PCR (TaqMan probes) and sequenced by Illumina MiSeq. Briefly, the adaptor reads and low‐quality reads were filtered from the raw data, the clean data was used in further alignments. Subsequently, the clean data was aligned to the human IGH database and analyzed using the online IMGT/HighV‐QUEST tool. The data included V, J assignment, CDR3 length distribution, clustering and other analyses.

## 3 RESULT

### 3.1 Quality control of all sequencing data

Using high‐throughput NGS, we sequenced repertoires CD4^+^ cells, CD8^+^ cells and tissue samples of nine pulmonary tuberculosis patients and nine normal controls. All data passed the QC process with an average Q20 >99.99%, Q30 >97.04%. A total sequencing data of total reads number (34094942), immune sequences number (33504380), unknown sequences number (590562), productive sequences number (24345651), nonproductive sequences number (9158729), *In‐frame* sequences number (27088243), *out‐of‐frame* sequences number (6326420), total CDR3 sequences number (23670829), Unique CDR3 nucleotide sequences number (1995707) and Unique CDR3 amino acids sequences number (1723256), details including data from each sample are listed in Table 1.

**Table 1:** Sequence quality of CD4^+^, CD8^+^, tissue sample of tuberculosis patients and normal controls

	M1‐CD4	M1‐CD8	M1‐Tissue	M2‐CD4	M2‐CD8	M2‐Tissue	M3‐CD4	M3‐CD8	M3‐Tissue	M4‐CD4	M4‐CD8	M4‐Tissue	M5‐CD4	M5‐CD8	M5‐Tissue	M6‐CD4	M6‐CD8	M6‐Tissue	M7‐CD4	M7‐CD8	M7‐Tissue	M8‐CD4	M8‐CD8
Total reads number	582217	459996	677310	903177	575955	335774	889156	643710	573785	1065180	859602	253607	889229	427415	495999	971852	627698	336292	776087	801797	313179	1016582	892231
Immune sequences number	550596	449546	650380	901272	573975	288998	887379	640989	539209	1062359	857908	195758	886866	424561	458664	970694	625998	286683	774495	799883	256256	1014869	890412
Unknown sequences numebr	31621	10450	26930	1905	1980	46776	1777	2721	34576	2821	1694	57849	2363	2854	37335	1158	1700	49609	1592	1914	56923	1713	1819
Productive sequences number	353828	250365	474701	699906	433221	162872	685579	492654	356619	841185	670586	98480	691340	327224	314326	764656	413577	82356	611495	700658	102129	810266	738949
Nonproductive sequences number	196768	199181	175679	201366	140754	126126	201800	148335	182590	221174	187322	97278	195526	97337	144338	206038	212421	204327	163000	99225	154127	204603	151463
In‐frame sequences number	377268	289584	502949	746867	461590	187810	731817	527307	377228	900754	717545	110971	738864	344971	323396	816839	435942	88467	654902	743398	106464	861847	783627
Out‐of_frame sequences number	163201	158365	145359	153605	111898	94557	154889	113184	159463	160716	139871	81018	147303	78997	129220	153394	189766	178497	119143	55925	143195	152559	106321
Total CDR3 sequences number	327059	233666	441542	677038	420582	153780	674805	484420	347379	817310	622440	84721	680369	321140	304849	752939	407462	46143	602093	691660	75485	798558	728731
Unique CDR3 nt sequences number	14090	12645	20153	60934	26558	16661	65880	32174	18011	65934	51955	11956	51934	18470	6677	123828	18610	4807	69335	22394	4851	74991	48370
Unique CDR3 aa sequences number	11656	10237	17021	51459	21942	15302	56729	27010	15661	54642	43502	11351	43869	15337	5265	112096	15068	4416	60466	18165	4371	63893	40937
Highly expressing clone number all	41	38	53	2	18	28	5	16	48	0	11	21	4	20	26	2	24	40	1	11	30	0	8
Highly expressing clone ratio all	0.68255575	0.52631534	0.63128989	0.02411238	0.31779772	0.71275849	0.05190685	0.34143305	0.68128471	0	0.15724086	0.6742012	0.05387518	0.50208009	0.90383436	0.05658493	0.4683357	0.7194374	0.00541112	0.6621259	0.78404981	0	0.30130597
Shannon entropy all	0.39719054	0.45082821	0.41825992	0.68833416	0.54280623	0.43039658	0.67979693	0.51286718	0.40022774	0.66933299	0.63017905	0.41873046	0.59746329	0.42595744	0.26421886	0.76166681	0.45595582	0.45273164	0.72728526	0.30304497	0.37782448	0.69998144	0.53009339

	M8‐Tissue	M9‐CD4	M9‐CD8	M9‐Tissue	N1‐CD4	N1‐CD8	N2‐CD4	N2‐CD8	N3‐CD4	N3‐CD8	N4‐CD4	N4‐CD8	N5‐CD4	N5‐CD8	N6‐CD4	N6‐CD8	N7‐CD4	N7‐CD8	N8‐CD4	N8‐CD8	N9‐CD4	N9‐CD8	Sum
Total reads number	345124	920454	612229	644546	734122	1015119	487365	1090624	948621	1738242	609,319	790,880	691,014	675,691	528,099	811,278	1,360,226	1,129,827	1,023,104	569,829	907,554	1,093,845	34,094,942
Immune sequences number	263278	918689	609826	615559	731341	1005773	486477	1089108	945180	1733207	598,136	778,657	689,656	671,243	526,754	806,631	1,355,308	1,122,850	1,015,052	558,523	905,671	1,089,711	33,504,380
Unknown sequences numebr	81846	1765	2403	28987	2781	9346	888	1516	3441	5035	11,183	12,223	1,358	4,448	1,345	4,647	4,918	6,977	8,052	11,306	1883	4,134	590,562
Productive sequences number	29271	740140	365631	448227	572690	632787	392543	748995	771280	478426	461,805	655,143	543,636	540,734	421,049	459,848	1,144,179	892,295	785,584	447,734	751,448	985,234	24,345,651
Nonproductive sequences number	234007	178549	244195	167332	158651	372986	93934	340113	173900	1254781	136,331	123,514	146,020	130,509	105,705	346,783	211,129	230,555	229,468	110,789	154,223	104,477	9,158,729
In‐frame sequences number	31239	791611	389714	478815	611479	674818	419284	803236	826442	1639631	491,438	700,926	581,739	579,260	447,370	493,172	1,214,667	940,753	837,153	476,306	801,996	1,026,787	27,088,243
Out‐of_frame sequences number	223550	126583	219578	132408	119590	330324	67051	285651	118315	93141	105,943	76,022	107,717	91,455	79,183	312,915	140,233	181,609	177,241	81,689	103,394	62,382	6,326,420
Total CDR3 sequences number	4069	729342	359173	437648	562036	620952	386428	737296	758769	471122	452,601	637,937	526,892	524,563	414,324	451,689	1,122,330	875,610	772,434	440,452	733,117	957,874	23,670,829
Unique CDR3 nt sequences number	2560	59322	22290	23141	58045	59010	47160	63725	123378	36185	27,333	27,072	117,546	33,206	87,434	39,496	52,585	27,685	57,173	28,979	127,052	34,112	1,995,707
Unique CDR3 aa sequences number	2529	49316	18368	20092	49690	51530	41236	55749	110754	30566	22,684	21,878	108,153	27,439	80,135	33,719	42,691	21,744	48,099	23,821	115,038	27,630	1,723,256
Highly expressing clone number all	14	0	7	47	0	5	3	10	4	14	15	10	4	12	2	10	7	16	3	12	3	10	655
Highly expressing clone ratio all	0.16515114	0	0.24276881	0.72208259	0	0.09839569	0.03277972	0.33245806	0.04359034	0.21227835	0.24436535	0.49173821	0.07930088	0.31473055	0.01548547	0.24206478	0.41441287	0.64089949	0.10992525	0.21462044	0.06475638	0.61016585	14.5499069
Shannon entropy all	0.8638929	0.66876197	0.52750687	0.40509952	0.67976672	0.59714855	0.7164873	0.55144703	0.74832534	0.57724095	0.49459581	0.37677137	0.78381492	0.52494136	0.79603452	0.58333764	0.44022788	0.34205038	0.5889533	0.56018664	0.75464691	0.33202264	24.7484339

### 3.2 Diversity and stability of repertoire in different groups

**Figure 1:**
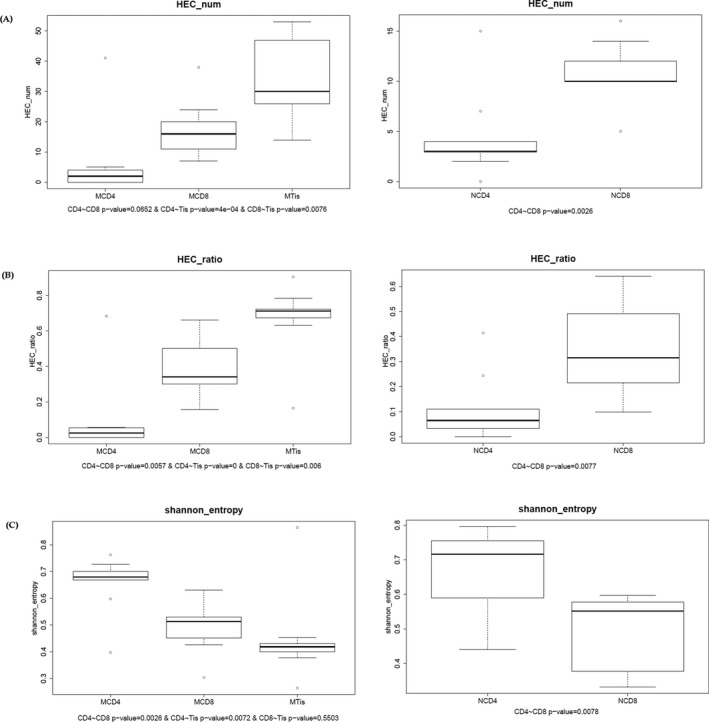
CDR3 clones in patients and controls. (a) HEC number comparison of patients and controls. MCD4: CD4^+^ cells of tuberculosis patients, MCD8: CD8^+^ cells of patients, MTis: tissue samples of patients. NCD4: CD4^+^ cells of controls, NCD8: CD8^+^ cells of controls. (b) HEC ratio analysis of patients and controls. (c) Shannon entropy of patients and controls

The distribution characteristics of the sequences and clone expansion were analyzed firstly. In the current study, the expression level of certain CDR3 clones higher than 0.5% of total clones was defined as high expansion clones (HECs). In tuberculosis patients, the HEC number was higher in the tissue group than that in the CD8 group or the CD4 group, while the comparison between the CD4 and CD8 groups showed no statistical difference. In the control groups, the HEC number in the CD8 group was significantly higher than that of the CD4 group (Figure 1a). In the comparison of HEC ratio, tuberculosis patients’ tissue group showed higher ratio than CD8 or CD4 groups, and that of CD8 group was higher than in CD4 group. In consistent with HEC number, the HEC ratio of CD8 group was higher than in CD4 group in control group (Figure 1b). The Shannon entropy measures multiplex of the immune system. It ranges from 0 to 1, “1” represents the most diversity and “0” represents the least diversity of immune system. In tuberculosis patients, Shannon entropy in CD4 was higher than CD8 or tissues, while tissue group showed the lowest Shannon entropy, although entropy of CD8 was not statistically higher than tissue group. In controls, Shannon entropy in CD4 was also higher than that of CD8 group (Figure 1c).

**Figure 2:**
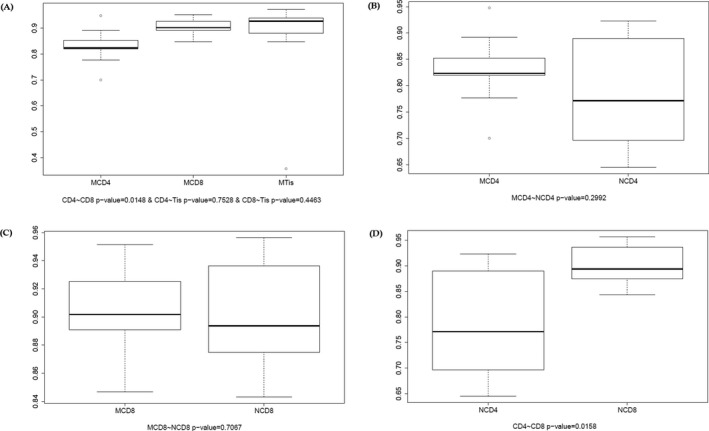
Gini coefficient of patients and controls. (a) Comparison of CD4^+^ cell group, CD8^+^ cell group and tissue group of patients. (b) Comparison of CD4^+^ cell group in patients and CD4^+^ cell group in controls. (c) Comparison of CD8^+^ cell in patient group and CD8^+^ group in control. (d) Comparison of control CD8^+^ cell group and CD4^+^ cell group

The Gini coefficient was then calculated to further understand the stability of tuberculosis patients’ immune system. In patients, Gini coefficient in CD8 was higher than in CD4 group, while other comparisons showed no significant change. In controls, Gini coefficient in CD8 group was higher than that of CD4 group (Figure 2).

### 3.3 CDR3 length distribution mode analysis

**Figure 3:**
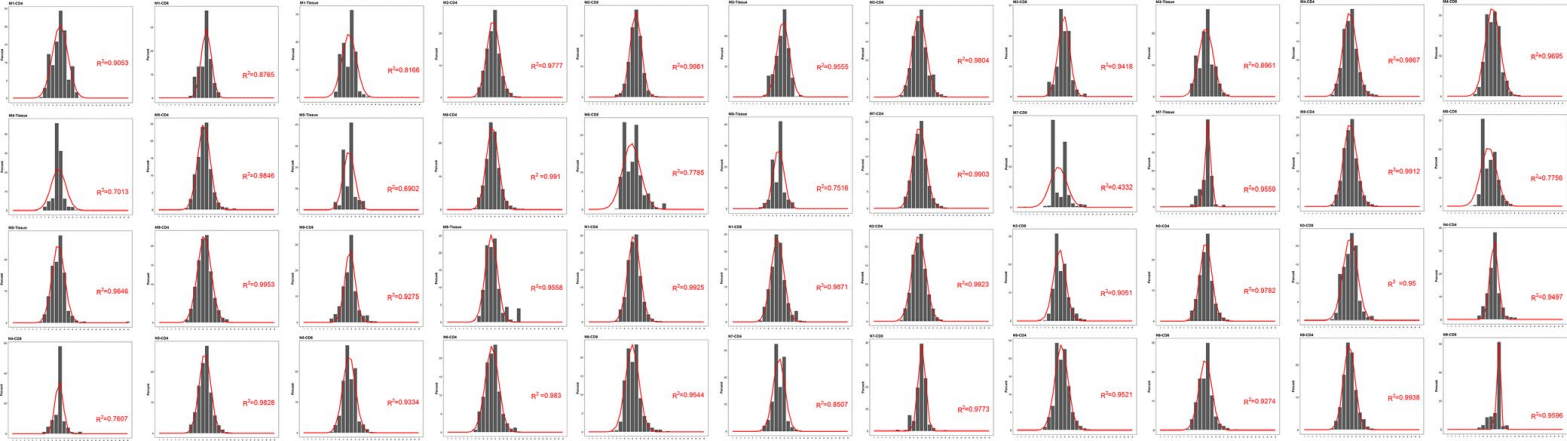
Gaussian distribution of *R* ^2 ^value in all samples. The X‐axis depicts each CDR3 length (1–30), and the Y‐axis depicts the total percentage of each length

**Figure 4:**
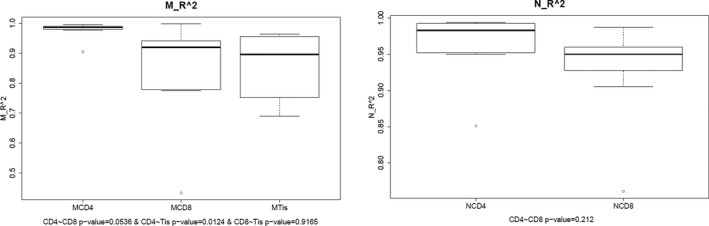
Gaussian distribution of *R* ^2 ^value between groups. (a) Comparison among CD4^+^, CD8^+^ and tissue sample groups in patients. (b) Comparison among CD4^+^ and CD8^+^ cell groups in controls

In addition, we made further analysis of CDR3 length distribution in all samples and the differences between groups. We first fit the Gaussian distribution of each sample and compared the R^2 ^value between each sample and each group. The R^2^ value ranged from 0 to 1, suggesting the worst fitted Gaussian distribution to the best fitted distribution. According to the R^2^ value, the length distribution of all samples was fitted to Gaussian distribution, although no statistical significance was found for comparing between groups, as shown in Figures 3 and 4.

**Figure 5:**
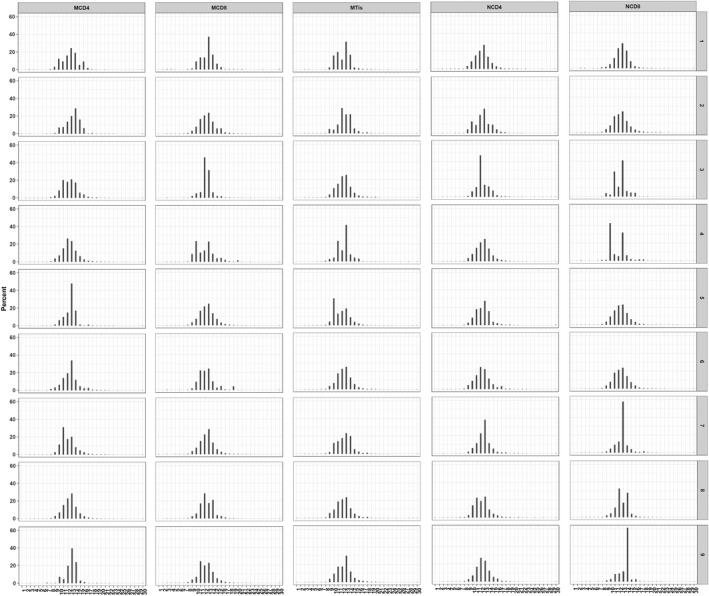
CDR3 length distribution in all samples. X‐axis represents length distribution, Y‐axis depicts the percentage of sequences of the corresponding length

**Figure 6:**
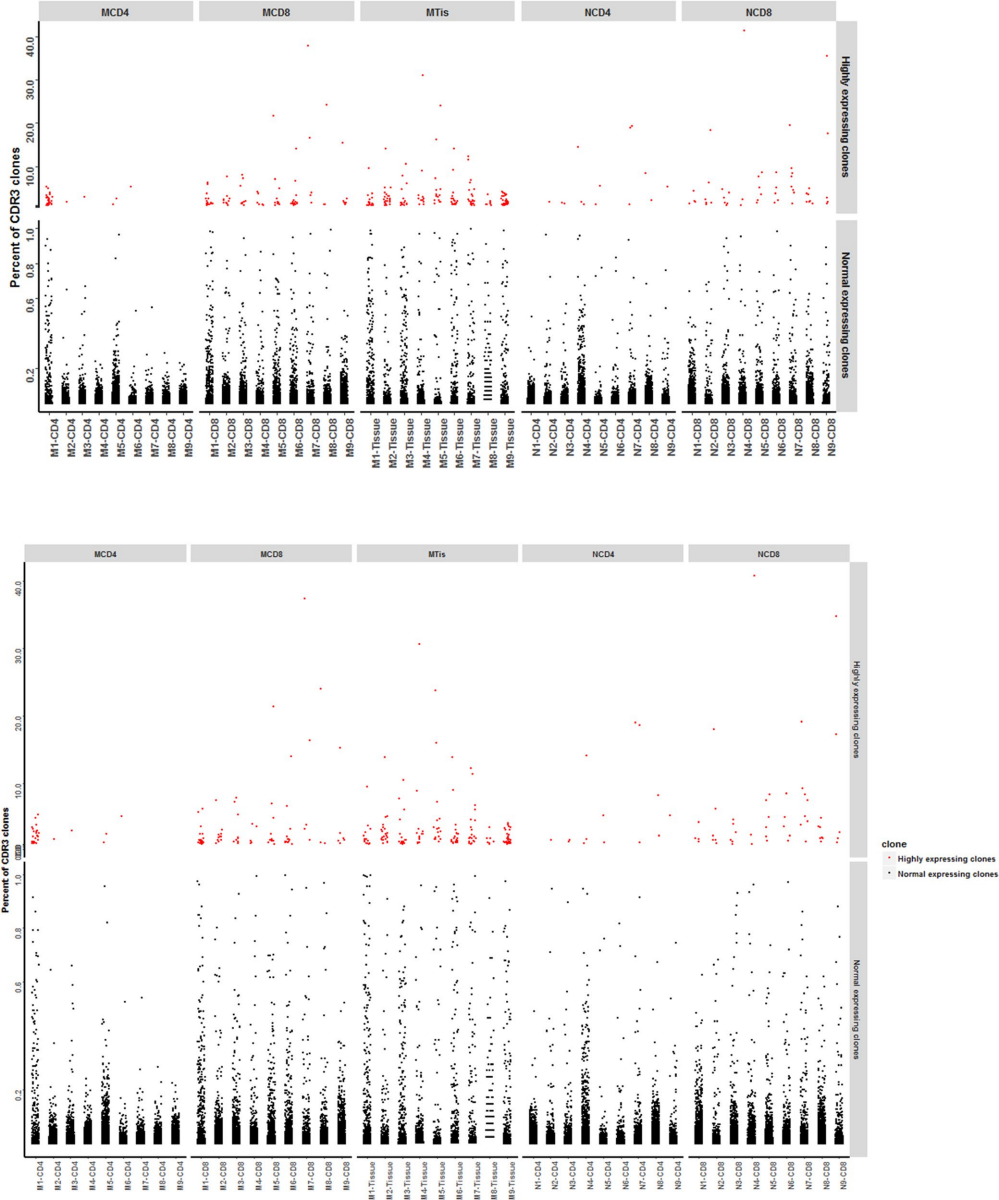
CDR3 expression status in all samples. X‐axis is sample ID and M1‐M9 represents tuberculosis patient number 1‐9. N1‐N9 represent control sample number 1‐9. CD4, CD8 and Tissue mean different sample types. Y‐axis is the percentage of CDR3 clones in each sample's different sequences. Red dots mean highly expressing clones (≥0.5%) and black dots represent normal expressing clones (<0.5%). (a) CDR3 clones (b) amino acid of CDR3 clones

The nucleotides and amino acids length of all samples were analyzed. As shown in Figures 3 and 5, the length distribution of CDR3 sequences ranged from 1–30 nucleotides and followed a Gaussian distribution. Besides, both nucleotides and amino acids length distribution of the high expression clones showed a significant difference between the control and tuberculosis patients. In both tuberculosis patients and controls, the amino acids sequence ranged from 1 to 30 amino acids and the highest percentage for both was 13 amino acid sequences. The CDR3 length of CD4, CD8 and tissue groups was analyzed, and it was observed that all presented with a similar pattern with the whole patient group. However, we found that the amino acid length of 1, 2, 5, 25, 27, 28, 29 were absent in more than seven samples in tissue group (*n* = 9), which is rare in CD4 and CD8 groups. In healthy controls, the amino acids sequence also ranged from 1 to 30 amino acids and the highest percentage was 13 amino acid sequences. For the distribution of CDR3 length, there were no statistically significant differences as has been found between groups. All samples showed a Gaussian distribution, the highest percentage centralized at 13 amino acids. Besides, CDR3 length of tissue group showed more skewed as length 1, 2, 5, 25, 27, 28, 29 were absent in most of tissue sample (Figure 6).

### 3.4 CDR3 sequence sharing modes analysis

Different individuals sharing an identical TCR sequence corresponding to the same antigenic epitope, termed public T cell response, were observed in a variety of immune responses, including tumorigenesis, autoimmunity, and viral infections. So, we counted the public T cell clones in each group based on the nucleotide and amino acid sequences of CDR3 (Li, Ye, Ji, & Han, 2012). In order to understand the immunological reaction to the common tuberculosis pathogens, the sharing pattern of CDR3 sequence were analyzed between patients. According to the sequence data, there were 586,248 nucleotide sequences and 504,126 amino acids sequences in CD4 group of patients, and 697,706 nucleotide sequences and 618,480 amino acids sequences in CD4 group of controls. There were 253,466 nucleotide sequences and 210,566 amino acids sequences in CD8 group of patients, 349,470 nucleotide sequences and 294,076 amino acids sequences in CD8 group of control. In addition, in tissue samples of patients, we obtained 108,817 nucleotide sequences and 96,008 amino acids sequences.

**Figure 7:**
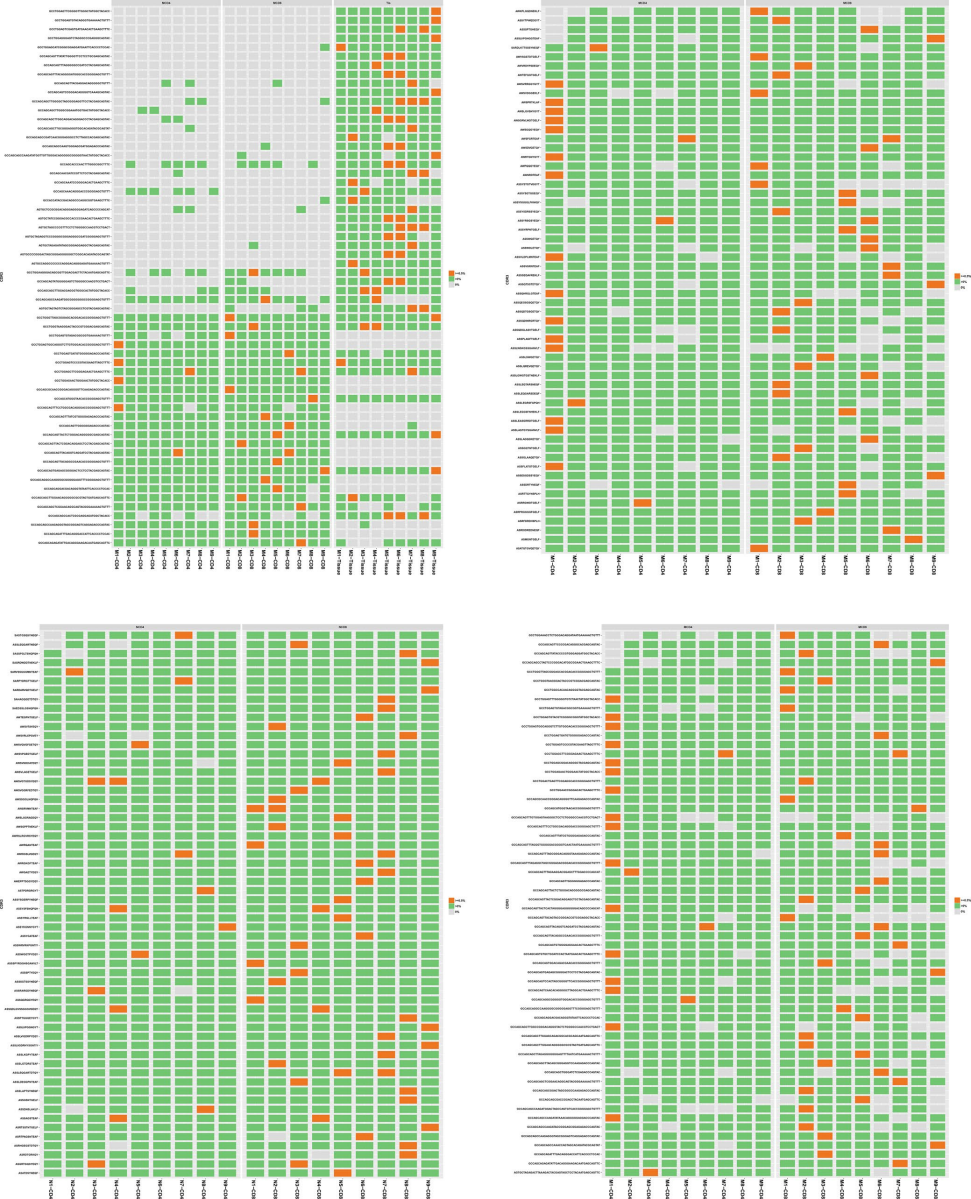
Top 60 expressing clones in all samples. Red rectangle: expression percentage ≥0.5%, green rectangle: expression percentage >0, gray rectangle = 0. (a) Nucleotide sequences of top 60 expressed clones in CD4^+^ and CD8^+^ group of patients. (b) Nucleotide sequences of top 60 expressed clones in CD4^+^, CD8^+^ and tissue group of patients. (c) Amino acids sequences of top 60 expressed clones in CD4^+^ and CD8^+^ group of patients. (d) Nucleotide sequences of top 60 expressed clones in CD4^+^ and CD8^+^ group of controls.

To elucidate the characteristics of sharing sequences, we compared the amino acid sequences and nucleotide sequences of highly expressed clones which were expressed in more than 0.5% in either patient group or control group (Figure 7). In patient group, eight amino acid sequences and eight nucleotide sequences were shared in all samples from CD4, CD8 and tissue groups. However, 35 amino acid sequences and 33 nucleotide sequences were shared in CD4 and CD8 samples of patient, while there were 61 amino acid sequences and 61 nucleotide sequences that were shared in CD4 and CD8 samples of control. No amino acid or nucleotide sequences were shared in CD4 and CD8 in both patients and controls. All shared sequences are displayed in Table 2.

**Table 2:** Shared sequence in all patient samples, in CD4^+^ and CD8^+^ cells of patients, in CD4^+^ and CD8^+^ cells of controls

Group	Shared sequences (NT)	Shared sequences (AA)
Shared sequences in all patients	GCCTGGAGCTTCGGGAGAACTGAAGCTTTC	AWSFGRTEAF
	GCCAGCATGGGTAACACCGGGGAGCTGTTT	ASMGNTGELF
	GCCAGCAGTTACTCTGGGACAGGGGGCGAGCAGTAC	ASSYSGTGGEQY
	GCCAGCAGTGAGAGCGGGGACTCCTCCTACGAGCAGTAC	ASSESGDSSYEQY
	GCCTGGGTAAGGGACTACCCGTCGGACGAGCAGTAC	AWVRDYPSDEQY
	GCCTGGAGTCCCCGTACGAAGTTAGCTTTC	AWSPRTKLAF
	GCCTGGGTTAGCGGGAGCACGGACACCGGGGAGCTGTTT	AWVSGSTDTGELF
	GCCAGCAGTGTCGGGACTCTCATCAATGAGCAGTTC	ASSVGTLINEQF
CD4 and CD8 of patient	GCCTGGAGCTTCGGGAGAACTGAAGCTTTC	AWSFGRTEAF
	GCCAGCATGGGTAACACCGGGGAGCTGTTT	ASMGNTGELF
	GCCAGCAGTTACTCTGGGACAGGGGGCGAGCAGTAC	ASSYSGTGGEQY
	GCCAGCAGAGATATTGACAGGGAAGACAATGAGCAGTTC	ASRDIDREDNEQF
	GCCAGCAGTGAGAGCGGGGACTCCTCCTACGAGCAGTAC	ASSESGDSSYEQY
	GCCTGGAGTGATGTGGGGGAGACCCAGTAC	AWSDVGETQY
	GCCAGCAGCCAAGAGGGTAGCGGGAGTCAGGAGACCCAGTAC	ASSQEGSGSQETQY
	GCCAGCAGTTACTCGGACAGGAGCTCCTACGAGCAGTAC	ASSYSDRSSYEQY
	GCCAGCAGATTTGACAGGGACCATTCACCCCTCCAC	ASRFDRDHSPLH
	GCCAGCAGTTACAGGCCGAACACCGGGGAGCTGTTT	ASSYRPNTGELF
	GCCAGCAGTTGGGGGGAGACCCAGTAC	ASSWGETQY
	GCCTGGGTTAGCGGGAGCACGGACACCGGGGAGCTGTTT	AWVSGSTDTGELF
	GCCAGCAGTTACAGGTCAGGATCCTACGAGCAGTAC	ASSYRSGSYEQY
	GCCAGCGCAACCGGGACAGGGGTTCAAGAGACCCAGTAC	ASATGTGVQETQY
	GCCTGGGTAAGGGACTACCCGTCGGACGAGCAGTAC	AWVRDYPSDEQY
	GCCTGGAGAACTGGGAACTATGGCTACACC	AWRTGNYGYT
	GCCAGCAGGCCAAGGGGCGGGGGAGGTTTCGGGGAGCTGTTT	ASRPRGGGGFGELF
	GCCTGGAGTGGCAGGGTCTTGTGGGACACCGGGGAGCTGTTT	ASSSEQAVREKLF
	GCCAGCAGCTCGGAACAGGCAGTACGGGAAAAACTGTTT	AWSGRVLWDTGELF
	GCCAGCAGTTTATCGTGGGGAGAGACCCAGTAC	ASSLSWGETQY
	GCCAGCAGTGTGGGGAGGAACACTGAAGCTTTC	ASSVGRNTEAF
	GCCAGCAGCTTGGAGCAGACGGCACGCAGCAATGAGCAGTTC	ASSLGWGTGSTNEKLF
	GCCTGGAGTTTGGGGGTGTCTAACTATGGCTACACC	ASSLEQTARSNEQF
	GCCTGGAGCGGACAGGGCTACGAGCAGTAC	AWSLGVSNYGYT
	GCCAGCAGGCGGGGGTGGGACACCGGGGAGCTGTTT	AWSGQGYEQY
	GCCTGGAACCGGGACACTGAAGCTTTC	ASRRGWDTGELF
	GCCTGGAGTCCCCGTACGAAGTTAGCTTTC	AWNRDTEAF
	GCCAGCAGCTTAGAGGGGGGGAGTTTTAATCATGAAAAACTGTTT	AWSPRTKLAF
	GCCTGGACTGAGTTCGGAGGCACCGGGGAGCTGTTT	ASSLEGGSFNHEKLF
	GCCAGCAGTTTACGTCCCGTTGAGGTCAATGAGCAGTTC	AWTEFGGTGELF
	GCCTGGAGTCTCCGGACAGGGTTCTGGGGGCAGGGCGCGGGTACGGGAGAGACCCAGTAC	ASSLEQGARSDEQF
	GCCAGCAGTTACGGGCGGGAGAAGTCCGGGGAGCTGTTT	ASSLRPVEVNEQF
	GCCAGCAGTTTCTCTCTAAACACCGGGGAGCTGTTT	AWSLRTGFWGQGAGTGETQY
		ASSYGREKSGELF
		ASSFSLNTGELF
CD4 and CD8 of controls	GCCTGGAGTGTAGGGACAGGGGACTCCTACGAGCAGTAC	AWSVGTGDSYEQY
	AGTGCTAGAGATGCGAGGGTTCAAGACACCGGGGAGCTGTTT	SARDARVQDTGELF
	GCCTGGAGTCGAGTGATGAACACTGAAGCTTTC	AWSRVMNTEAF
	GCCTGGAGGATAGGGAGTCTAGTAGGCGAGCAGTAC	AWRIGSLVGEQY
	AGTGCTCATGCCGGACAGGGAGAAACAGATACGCAGTAT	SAHAGQGETDTQY
	AGTGCTAGACCTTACGACAGGGGGACCACCGGGGAGCTGTTT	SARPYDRGTTGELF
	AGTGCTAGTCGGGATTGGGACGATACTAATGAAAAACTGTTT	SASRDWDDTNEKLF
	GCCAGCAGCTTGGAACAGGGGGCTCGCACAGATACGCAGTAT	ASSLEQGARTDTQY
	GCCTGGAGGGGAAAGGGTTTCACTGAAGCTTTC	AWSVLAGETGELF
	GCCTGGAGCGTCCTAGCGGGAGAAACCGGGGAGCTGTTT	AWRGKGFTEAF
	GCCAGCAGTTACAGGGGCCTATTGACTGAAGCTTTC	ASSYRGLLTEAF
	GCCAGCACTCCGGACAGGGGACGAGGCTACACC	ASTPDRGRGYT
	GCCAGCAGTTTAAAGGGACCTTACACTGAAGCTTTC	ASSLKGPYTEAF
	GCCAGCAGTTACTCAGGCGAGCGGCCCTACAATGAGCAGTTC	ASSYSGERPYNEQF
	GCCTGGAGTGTGACAGCCTACGAGCAGTAC	AWSVTAYEQY
	GCCTGGAGTGTACAGGTCGGGTTTGGAGAGACCCAGTAC	AWSVQVGFGETQY
	GCCAGCAGTTACAAGGGAAACAACTATGGCTACACC	ASSYKGNNYGYT
	GCCTGGGGGGCGGAGACCTACGAGCAGTAC	AWGAETYEQY
	GCTAGTGCCACAGACTCCTACAATGAGCAGTTC	ASATDSYNEQF
	GCCAGCAGTTACGGGGCTACTGAAGCTTTC	ASSYGATEAF
	GCCAGCAGTTTAGCACCCACCTCCTACAATGAGCAGTTC	ASSLAPTSYNEQF
	GCCAGCAGTTGGAGGGTAAGGAAGCCTGGAAACACCATATAT	ASSWRVRKPGNTIY
	GCCAGCAGCTTGGTGATCGGGGATCGCCCCTACGAGCAGTAC	ASSLVIGDRPYEQY
	GCCAGCAGCCAAGGAAGACAGGGCTACGAGCAGTAC	ASSQGRQGYEQY
	GCCAGCAGTTACAGTTTTAGCAATCAGCCCCAGCAT	ASSYSFSNQPQH
	GCCAGCAGTTCCCCCACCTACGAGCAGTAC	ASSSPTYEQY
	AGTGCCGAGGACAGTTCGTTGGGGAGCAATCAGCCCCAGCAT	SAEDSSLGSNQPQH
	GCCAGCAGTCCGACAGGGGGCGGGGAGTATGGCTACACC	ASSPTGGGEYGYT
	GCCTGGGAACCCCCGACTAGCGGGGGGTACGAGCAGTAC	AWEPPTSGGYEQY
	GCCAGTAGCGGGAGTAACACCGGGGAGCTGTTT	ASSGSNTGELF
	GCCTGGAGGAGTTTACGGGGCGTAAGGTCCTACGAGCAGTAC	AWRSLRGVRSYEQY
	GCCAGCAGGACTAGCTCCACGAACACCGGGGAGCTGTTT	ASRTSSTNTGELF
	GCCAGCAGTTTAGATGAGGGGGGGCCGAACACTGAAGCTTTC	ASSLDEGGPNTEAF
	GCCTGGAGCTCGGGAGGCCTCAATCAGCCCCAGCAT	AWSSGGLNQPQH
	GCCAGCGGGAGGACGGGACAGGGCTACGAGCAGTAC	ASGRTGQGYEQY
	GCCTGGACCGAGGGACCGAACACCGGGGAGCTGTTT	AWTEGPNTGELF
	AGTGCTAGAGTTTCTTCGGGTGGAGGGATGAACACTGAAGCTTTC	SARVSSGGGMNTEAF
	GCCAGCAGTTCTGGGACTAGCAGTTACAATGAGCAGTTC	ASSSGTSSYNEQF
	GCCTGGAGACAGGCGAACACTGAAGCTTTC	ASSLGTDRSTEAF
	GCCAGCAGCTTGGGGACAGATCGCTCGACTGAAGCTTTC	AWRQANTEAF
	GCCAGCAGCCTCGTGGGCGACAGACACTACTCTGGAAACACCATATAT	ASSLVGDRHYSGNTIY
	GCCAGCAGCTCGCCCTACAGGGGCGGCCACTCTGGGGCCAACGTCCTGACT	ASSSPYRGGHSGANVLT
	GCCAGCAGTGCAGGGTCCACTGAAGCTTTC	ASSAGSTEAF
	GCCTGGAGTGTTGGAGGGAGGTTTGAAGATACGCAGTAT	AWSVGGRFEDTQY
	GCCAGCAGCCAAGATCTCGGGGTTTCGTCAGGGGGAGGGGTGGGGGAGCAGTAC	ASSQDLGVSSGGGVGEQY
	GCCTGGAGTTTAAGCGGGAGGGCCGGCGAGCAGTAC	AWSLSGRAGEQY
	GCCTGGAGTGTACCAGGGGAGGACACCGGGGAGCTGTTT	AWSVPGEDTGELF
	GCCAGCAGTTTAGTCCCAGGGGGAAATGGCTACACC	ASSLVPGGNGYT
	GCCTGGAGTGGTCCCCCAACTAATGAAAAACTGTTT	AWSGPPTNEKLF
	GCCAGCAGTTGGGGGGGGACTCCCTACGAGCAGTAC	ASSWGGTPYEQY
	GCCAGCAGTCGCGGGGCAGGGGGTTACAATGAGCAGTTC	ASSRGAGGYNEQF
	GCCTGGAGTGAGGGGGTCGGAAACACCATATAT	AWSEGVGNTIY
	GCCTGGAGTTCGACAGGGTCTAGCACAGATACGCAGTAT	AWSSTGSSTDTQY
	GCCAGCAGTTATGGACAGTTCTCCATTCAGTAC	ASSYGQFSIQY
	GCCAGCAGCTCCTACTTCCGACAGGGCCCCCAAGAGACCCAGTAC	ASSSYFRQGPQETQY
	GCCAGCAGCAGTCATGGGGGGCGAGAGGACGAGCAGTAC	ASSSHGGREDEQY
	GCCTGGGCCGACAGCTCTGGAAACACCATATAT	AWADSSGNTIY
	GCCTGGAGGGACGGGGGGCGGGACACCGGGGAGCTGTTT	AWRDGGRDTGELF
	GCCAGCAGCTCGGGGACAGGGAGAGGGGATGGCTACACC	ASSSGTGRGDGYT
	GCCAGCAGCTTGGGCCCATTAGGGGCGGCTAACTATGGCTACACC	ASSLGPLGAANYGYT
	GCCAGCAGCTTGGGCCTACTAGCAGATACGCAGTAT	ASSLGLLADTQY

### 3.5 Significance of TRBV and TRBJ usage in patients and controls

In addition, to elucidate the potential specific immune reaction to tuberculosis, usage of TRBV and TRBJ was analyzed in all groups.

**Figure 8:**
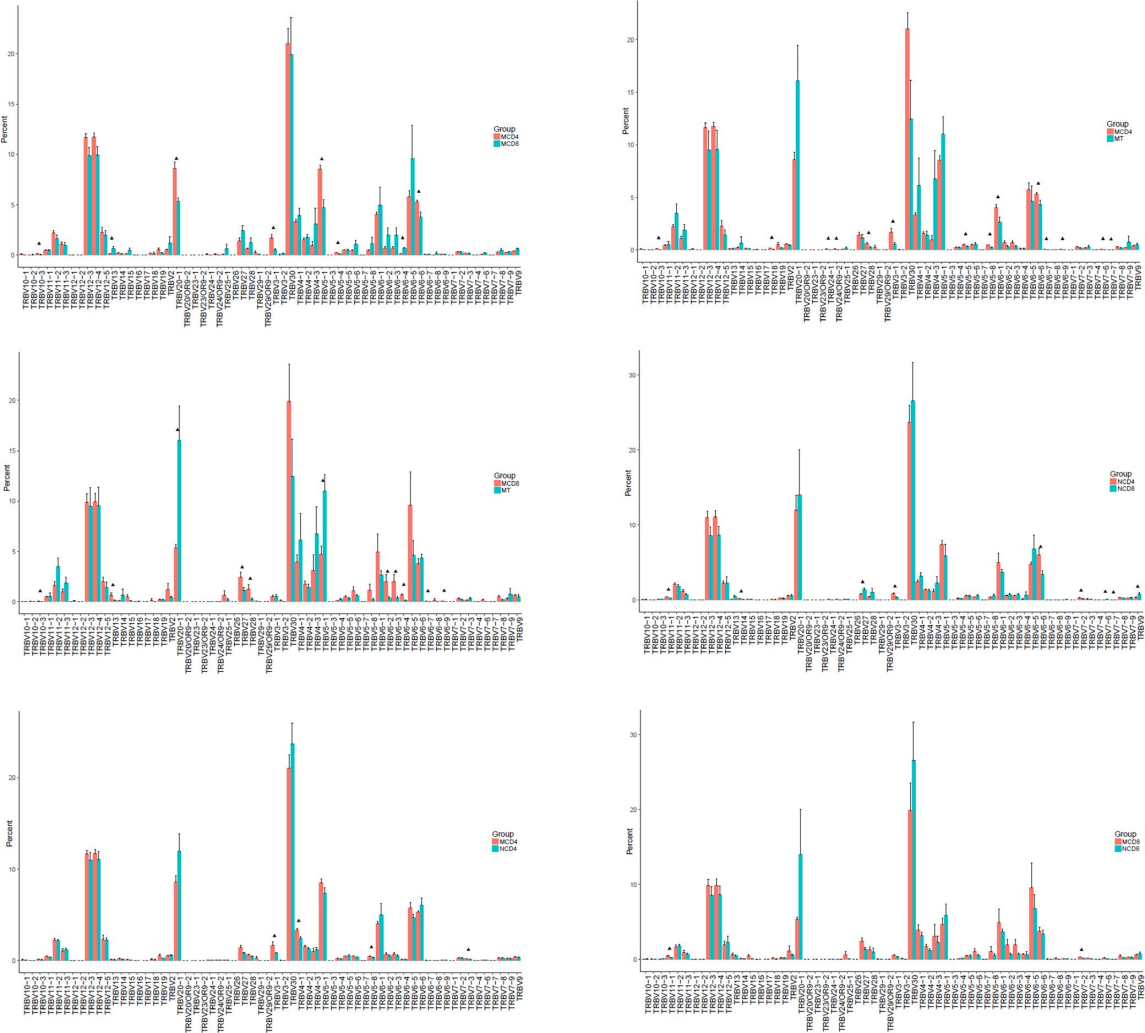
Comparison of TRBV gene usage in groups. X‐axis represents the genes of TRBV. Y‐axis represents the expressing percentage of corresponding gene: (a) CD4^+^ cell and CD8^+^ cell groups in patients. (b) CD4^+^ cell and tissue groups in patients. (c) CD8^+^ cell and tissue groups in patients. (d) CD4^+^ cell and CD8^+^ cell groups in controls. (e) CD4^+^ cell group of patients and CD4^+^ cell group of controls. (f) CD8^+^ cell group of patients and CD8^+^ cell group of controls

In patients, TRBV13 and TRBV6‐4 were significantly highly expressed in CD8 group than in CD4 group, on the contrary TRBV10‐3, TRBV20‐1, TRBV3‐1, TRBV5‐1, TRBV5‐4, TRBV6‐6 showed significantly lower expression in CD8 group than in CD4 group. The expression of TRBV10‐3, TRBV18, TRBV24‐1, RBV24/OR9‐2, TRBV28, TRBV3‐1, TRBV5‐5, TRBV5‐8, TRBV6‐1, TRBV6‐6, TRBV6‐7, TRBV6‐9, TRBV7‐6, TRBV7‐7 was also higher in CD4 than in tissue group. TRBV10‐3, TRBV13, TRBV27, TRBV28, TRBV6‐2, TRBV6‐3, TRBV6‐4, TRBV6‐7, TRBV6‐9 were highly expressed in CD8 samples than in tissues while TRBV20‐1, TRBV5‐1 showed lower expression in CD8 samples than in tissue samples. In controls, TRBV11‐1, TRBV14, TRBV3‐1, TRBV6‐6, TRBV7‐2 and TRBV7‐7 exhibited significant higher expression in CD4 group than in CD8 group, while TRBV27, TRBV7‐6, TRBV9 all showed lower expression in CD4 group than in CD8 group. Moreover, TRBV3‐1 and TRBV4‐1, TRBV5‐8 and TRBV7‐3 in controls’ CD4 group showed statistically lower expression than that of in patients. TRBV11‐1 and TRBV7‐2 exhibited higher expression in patients’ CD8 group than that of control's (Figure 8).

**Figure 9:**
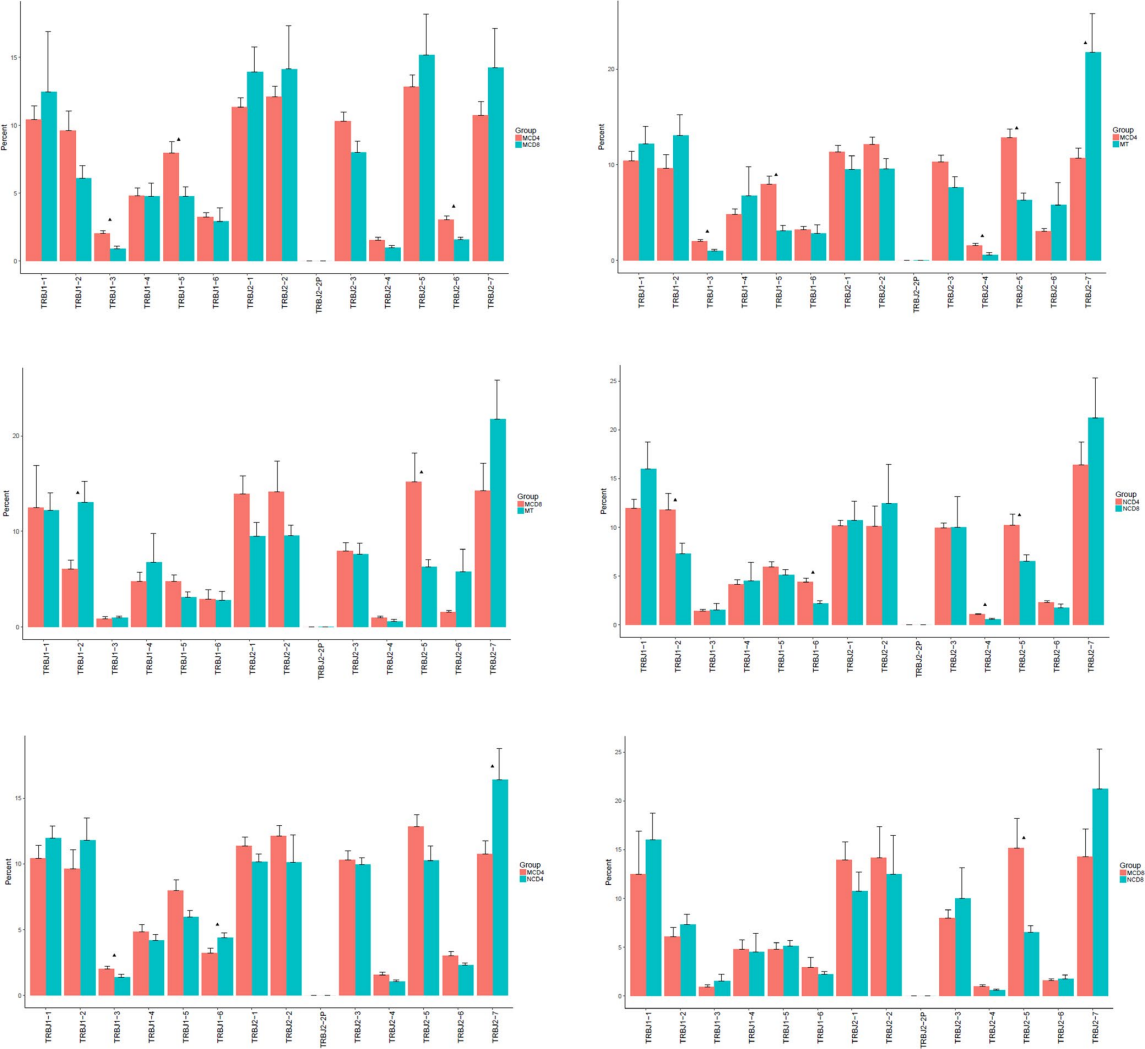
Comparison of TRBJ gene usage in groups. X‐axis represents the genes of TRBJ. Y‐axis depicts expressing percentage of corresponding gene. (a) CD4^+^ cell and CD8^+^ cell groups in patients. (b) CD4^+^ cell and tissue groups in patients. (c) CD8^+^ cell and tissue groups in patients. (d) CD4^+^ cell and CD8^+^ cell groups in controls. (e) CD4^+^ cell group of patients and CD4^+^ cell group of controls. (f) CD8^+^ cell group of patients and CD8^+^ cell group of controls

**Figure 10:**
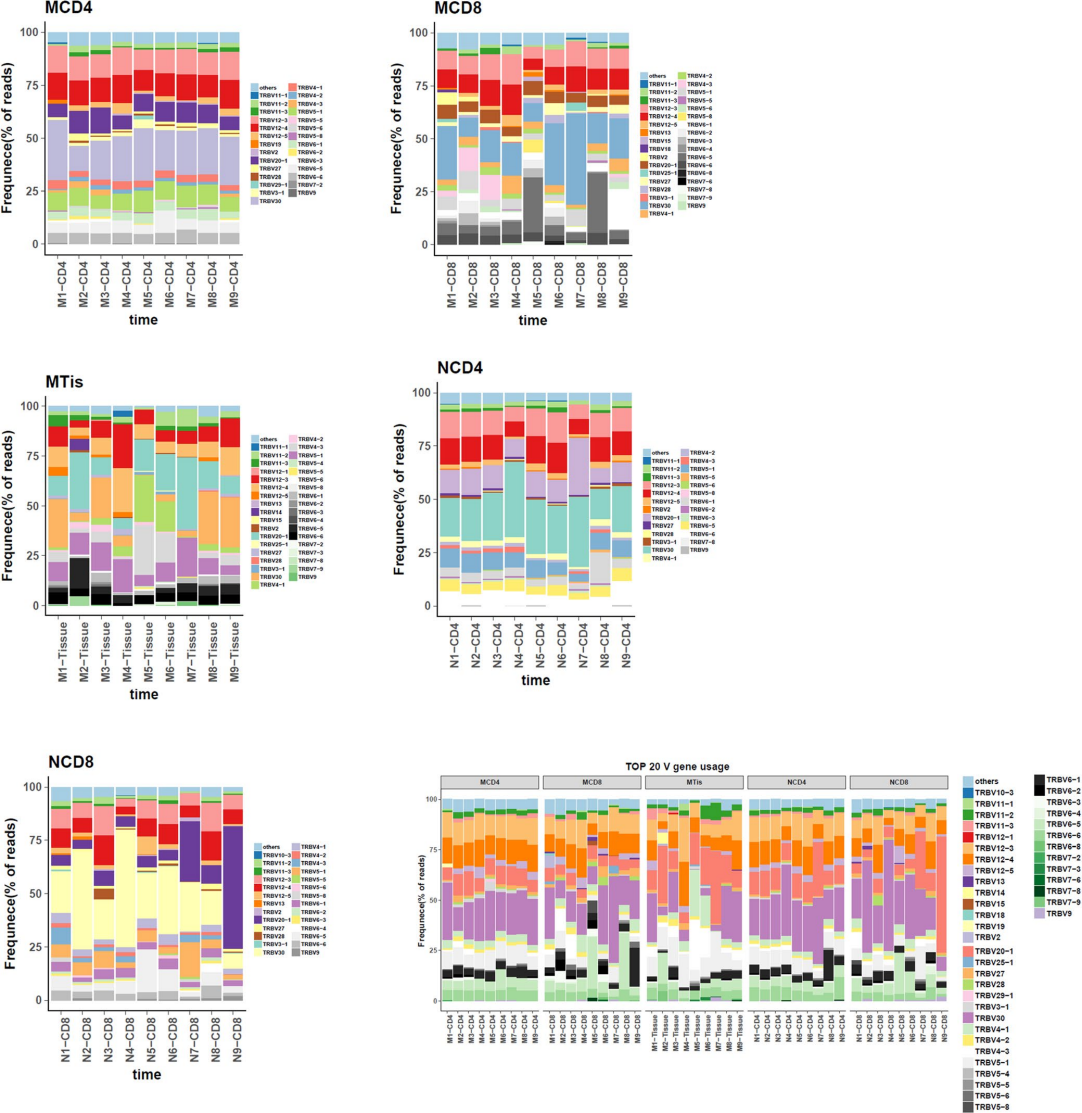
Top 20 used TRBV genes in each group. X‐axis is sample ID, N1‐N9 represent control sample number 1–9. CD4, CD8 and Tissue mean different sample types. Y‐axis represents the frequencies of corresponding reads. (a) Top 20 used TRBV genes in CD4^+^ cell group of patients. (b) Top 20 used TRBV genes in CD8^+^ cell group of patients. (c) Top 20 used TRBV genes in tissue group of patients. (d) Top 20 used TRBV genes in CD4^+^ cell group of controls. (e) Top 20 used TRBV genes in CD8^+^ cell group of controls. (f) Top 20 used TRBV genes in all sample groups

In patients, TRBJ1‐3, TRBJ2‐6 were significantly highly expressed in CD8 group than in CD4 group, on the contrary TRBJ1‐5 showed lower expression in CD8 group than in CD4 group. The expression of TRBJ1‐3, TRBJ1‐5, TRBJ2‐4, TRBJ2‐5 was also higher in CD4 than in tissue group, and TRBJ2‐7 exhibited lower expression in CD4 than in tissue group. TRBJ1‐2 showed lower expression and TRBJ2‐5 presented higher expression in CD8 group than in tissue group respectively. In controls, TRBJ1‐2, TRBJ1‐6, TRBJ2‐4, TRBJ2‐5 exhibited significant higher expression in CD4 group than in CD8 group. Besides, TRBJ1‐6 and TRBJ2‐7 in controls’ CD4 group showed statistical higher expression than that of patients. While TRBJ1‐3 in controls’ CD4 group showed lower expression than patients’ expression. TRBJ2‐5 exhibited higher expression in patients’ CD8 group than that of control's (Figure 9). Top 20 genes in each group are shown in Figure 10.

**Figure 11:**
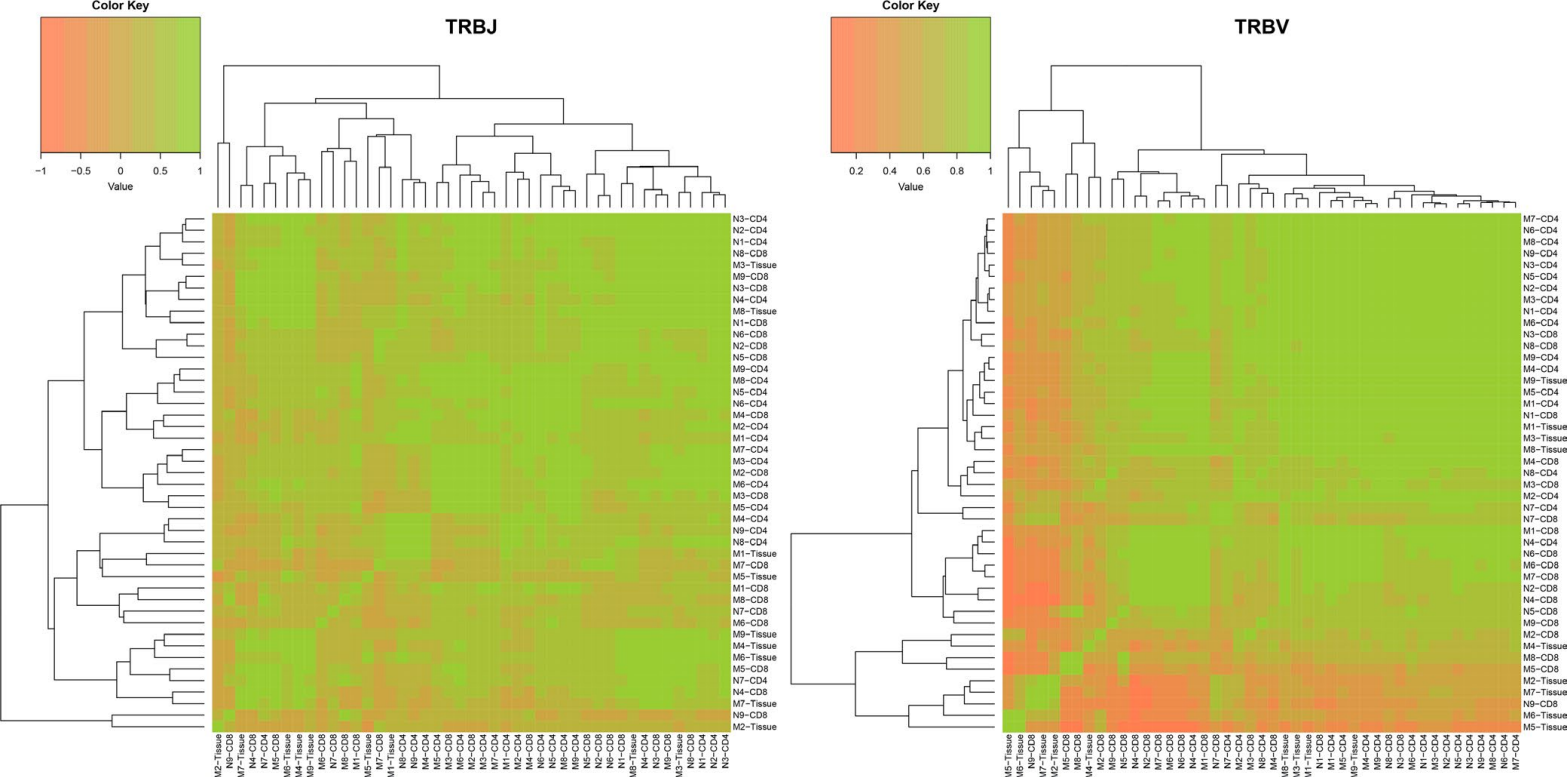
Heatmap of TRBV and TRBJ usage in all samples. Heatmap of gene usage in comparison of all samples for (a) TRBV (b) TRBJ

Additionally, we combined the expression data of all samples on TRBV or TRBJ to understand the correlation between the expression in the samples. The heatmaps are shown in Figure 11.

### 3.6 Combination of usage of TRBV and TRBJ in tuberculosis patients and control samples

TRBV/TRBJ combination was an important source of CDR3 sequence diversification. Within all TRBV/TRBJ combinations, we first counted the highly expressed which represent more than 0.5% of all combinations in each group. For CD4 group in controls, there were six TRBV/TRBJ combinations which were used more than 0.5%, and the number is 22 in CD8 group in controls. In the tuberculosis patient group, there were also six TRBV/TRBJ combinations which were used more than 0.5% in CD4 group, and 21 high expression TRBV/TRBJ in CD8 group, and 32 high expression TRBV/TRBJ in tissue group (Table 3).

**Table 3:** Combination of usage of TRBV and TRBJ in tuberculosis patients and control samples

V_type	J_type	count	individual	Percent
TRBV30	TRBJ2‐7	243,097	N7‐CD4	21.6600287
TRBV20‐1	TRBJ2‐2	233,941	N7‐CD4	20.8442259
TRBV30	TRBJ2‐7	77,626	N4‐CD4	17.1510889
TRBV30	TRBJ2‐5	51,206	N5‐CD4	9.71850019
TRBV6‐1	TRBJ1‐2	66,181	N8‐CD4	8.56785175
TRBV6‐6	TRBJ1‐2	49,082	N9‐CD4	6.69497502
TRBV30	TRBJ2‐7	285,440	N4‐CD8	44.7442302
TRBV20‐1	TRBJ2‐2	361,501	N9‐CD8	37.7399324
TRBV30	TRBJ1‐1	164,581	N2‐CD8	22.3222424
TRBV20‐1	TRBJ2‐3	190,384	N7‐CD8	21.7430134
TRBV20‐1	TRBJ1‐4	178,703	N9‐CD8	18.6562116
TRBV30	TRBJ1‐1	62,806	N6‐CD8	13.9046999
TRBV30	TRBJ2‐2	108,417	N7‐CD8	12.3818823
TRBV30	TRBJ2‐7	80,921	N2‐CD8	10.9753749
TRBV5‐1	TRBJ2‐3	87,949	N7‐CD8	10.044312
TRBV30	TRBJ2‐7	48,860	N5‐CD8	9.31441981
TRBV6‐5	TRBJ1‐1	48,079	N5‐CD8	9.16553398
TRBV12‐3	TRBJ1‐1	73,219	N7‐CD8	8.36205617
TRBV6‐5	TRBJ2‐1	41,462	N5‐CD8	7.90410303
TRBV12‐3	TRBJ2‐1	32,958	N8‐CD8	7.4827677
TRBV30	TRBJ2‐7	63,002	N7‐CD8	7.19521248
TRBV30	TRBJ1‐1	42,289	N1‐CD8	6.81034927
TRBV30	TRBJ2‐7	29,944	N6‐CD8	6.6293401
TRBV4‐3	TRBJ2‐7	36,285	N1‐CD8	5.84344684
TRBV12‐3	TRBJ1‐2	25,505	N8‐CD8	5.79064234
TRBV6‐5	TRBJ1‐1	25,641	N6‐CD8	5.67669348
TRBV12‐5	TRBJ2‐1	28,982	N5‐CD8	5.52497984
TRBV12‐3	TRBJ1‐1	24,064	N3‐CD8	5.10780647
TRBV30	TRBJ1‐2	45,654	M1‐CD4	13.9589493
TRBV12‐3	TRBJ2‐2	33,833	M1‐CD4	10.3446167
TRBV12‐3	TRBJ1‐1	24,993	M1‐CD4	7.64174048
TRBV6‐5	TRBJ2‐7	44,913	M6‐CD4	5.96502506
TRBV20‐1	TRBJ2‐1	39,180	M3‐CD4	5.80612177
TRBV30	TRBJ1‐1	17,997	M1‐CD4	5.50267689
TRBV30	TRBJ1‐1	281,888	M7‐CD8	40.7552844
TRBV6‐5	TRBJ2‐2	177,317	M8‐CD8	24.3322982
TRBV6‐5	TRBJ2‐7	68,199	M5‐CD8	21.2365324
TRBV12‐3	TRBJ2‐1	132,063	M7‐CD8	19.0936298
TRBV30	TRBJ2‐5	73,658	M6‐CD8	18.0772686
TRBV6‐1	TRBJ2‐7	55,800	M9‐CD8	15.5356889
TRBV4‐3	TRBJ2‐5	39,717	M3‐CD8	8.19887701
TRBV30	TRBJ2‐7	39,449	M3‐CD8	8.14355312
TRBV12‐3	TRBJ1‐6	38,230	M3‐CD8	7.89191198
TRBV6‐2	TRBJ2‐7	31,203	M2‐CD8	7.41900509
TRBV12‐3	TRBJ2‐5	43,944	M4‐CD8	7.05995759
TRBV6‐2	TRBJ2‐2	22,112	M5‐CD8	6.88547051
TRBV30	TRBJ2‐2	15,645	M1‐CD8	6.6954542
TRBV12‐3	TRBJ2‐5	27,212	M6‐CD8	6.67841418
TRBV6‐2	TRBJ2‐5	27,203	M6‐CD8	6.67620539
TRBV30	TRBJ1‐4	15,233	M1‐CD8	6.51913415
TRBV12‐3	TRBJ2‐2	30,744	M3‐CD8	6.34655877
TRBV12‐3	TRBJ2‐2	38,148	M4‐CD8	6.1287835
TRBV5‐1	TRBJ2‐1	24,851	M2‐CD8	5.90871697
TRBV2	TRBJ2‐5	12,950	M1‐CD8	5.54209855
TRBV5‐8	TRBJ1‐6	16,347	M5‐CD8	5.09030329
TRBV12‐3	TRBJ2‐7	27,534	M4‐Tissue	32.4996164
TRBV4‐3	TRBJ1‐2	73,434	M5‐Tissue	24.0886472
TRBV4‐1	TRBJ2‐6	54,498	M5‐Tissue	17.8770473
TRBV20‐1	TRBJ1‐4	27,013	M2‐Tissue	17.5660034
TRBV5‐1	TRBJ2‐7	13,014	M7‐Tissue	17.2405114
TRBV30	TRBJ1‐1	70,700	M1‐Tissue	16.0120668
TRBV4‐3	TRBJ1‐2	6,465	M6‐Tissue	14.0107925
TRBV20‐1	TRBJ2‐6	9,564	M7‐Tissue	12.6700669
TRBV20‐1	TRBJ2‐7	9,212	M7‐Tissue	12.2037491
TRBV5‐1	TRBJ1‐2	39,469	M3‐Tissue	11.3619419
TRBV6‐5	TRBJ1‐1	16,242	M2‐Tissue	10.5618416
TRBV4‐1	TRBJ2‐6	4,634	M6‐Tissue	10.0426934
TRBV5‐1	TRBJ1‐2	8,463	M4‐Tissue	9.98925886
TRBV12‐3	TRBJ1‐1	38,671	M9‐Tissue	8.83609659
TRBV30	TRBJ2‐7	29,333	M3‐Tissue	8.44409132
TRBV12‐3	TRBJ2‐2	22,954	M5‐Tissue	7.52962942
TRBV30	TRBJ2‐1	25,885	M3‐Tissue	7.45151549
TRBV12‐3	TRBJ2‐2	25,845	M3‐Tissue	7.44000069
TRBV5‐1	TRBJ2‐1	11,003	M2‐Tissue	7.15502666
TRBV30	TRBJ1‐6	30,986	M1‐Tissue	7.01767895
TRBV30	TRBJ1‐1	285	M8‐Tissue	7.00417793
TRBV30	TRBJ2‐7	285	M8‐Tissue	7.00417793
TRBV12‐3	TRBJ2‐7	29,615	M9‐Tissue	6.76685373
TRBV12‐3	TRBJ2‐7	19,908	M5‐Tissue	6.53044622
TRBV12‐3	TRBJ2‐7	4,895	M7‐Tissue	6.48473207
TRBV30	TRBJ2‐7	27,923	M9‐Tissue	6.38024166
TRBV30	TRBJ1‐4	26,309	M9‐Tissue	6.01145213
TRBV5‐1	TRBJ2‐7	2,719	M6‐Tissue	5.89255142
TRBV14	TRBJ2‐3	8,461	M2‐Tissue	5.50201587
TRBV20‐1	TRBJ1‐1	2,425	M6‐Tissue	5.25540169
TRBV5‐1	TRBJ2‐1	4,310	M4‐Tissue	5.08728651
TRBV11‐3	TRBJ2‐2	22,117	M1‐Tissue	5.00903651

**Figure 12:**
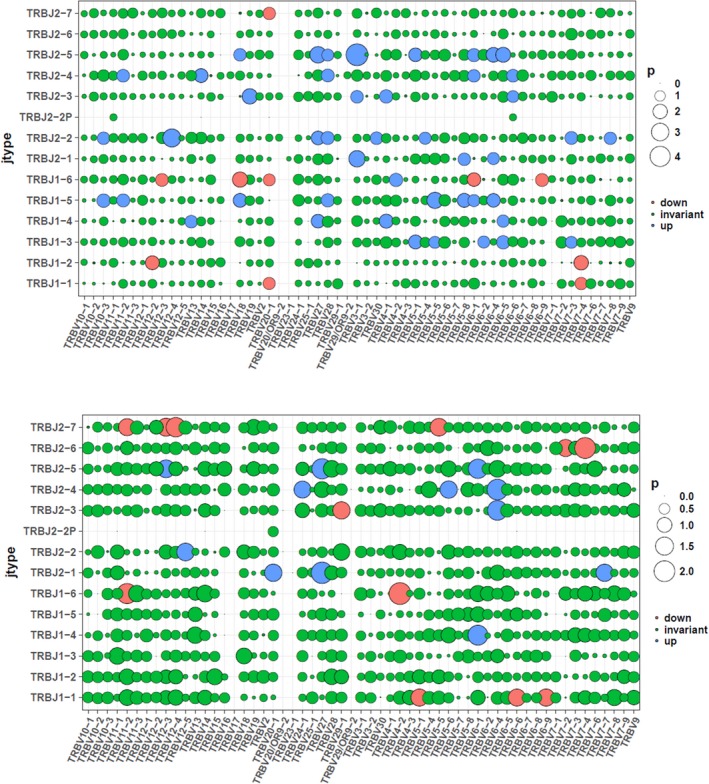
TRBV/TRBJ combination in groups. (a) Comparison between CD4^+^ cell group in patients and CD4^+^ cell group in controls. (b) Comparison between CD8^+^ cell group in patients and CD8^+^ cell group in controls

**Figure 13:**
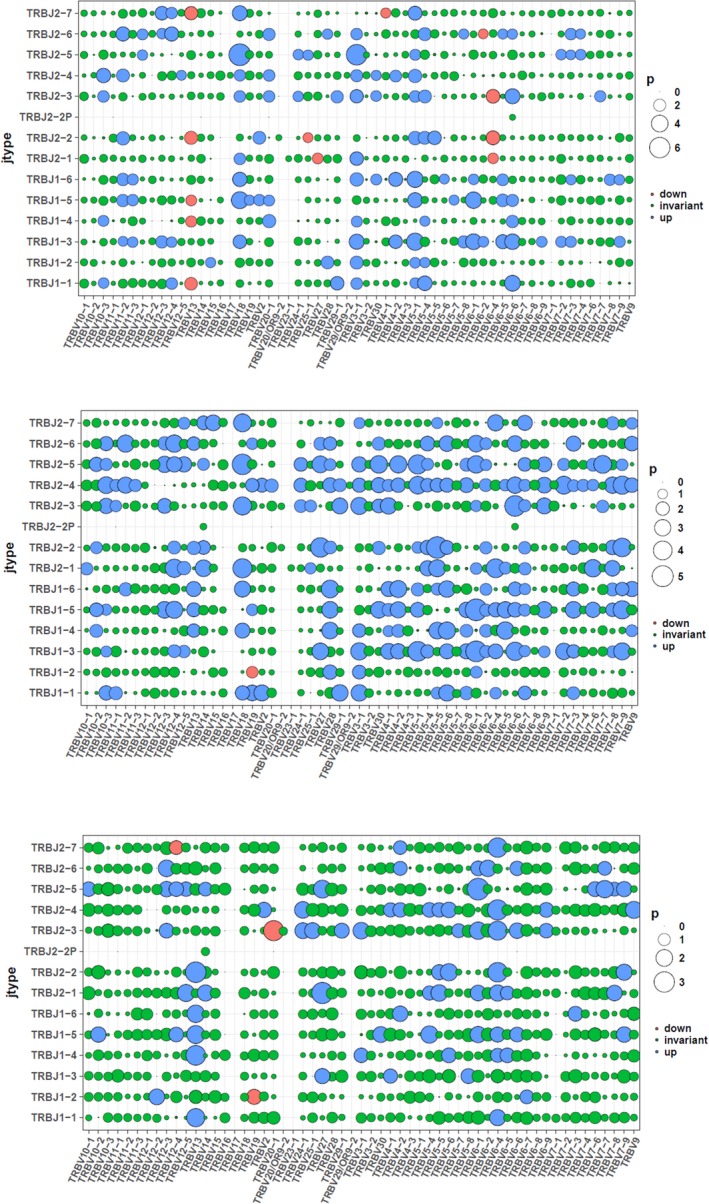
TRBV/TRBJ combination in groups. (a) Comparison between CD8^+^ cell group in patients and CD4^+^ cell group in patients. (b) Comparison between tissue group in patients and CD4^+^ cell group in patients. (c) Comparison between CD8^+^ cell group in patients and tissue group in patients

In order to examine the potential contribution of specific TRBV/TRBJ combinations to disease progress, comparison of the relative frequencies of TRBV/TRBJ combinations between patients and controls was performed. There were 46 up‐regulated and 10 down‐regulated TRBV/TRBJ combinations as has been found after comparison between CD4 group of patients and controls. There were 12 up‐regulated combinations and 12 down‐regulated combinations as has been found after comparison between CD8 group of patients and controls (Figure 12). We then compared different TRBV/TRBJ combinations in CD4, CD8, tissue group of patients as shown in Figure 13.

## 4 DISCUSSION

Tuberculosis, a well‐known infectious disease, is closely related to immune reaction in its development, diagnosis and treatment process (Janis, Kaufmann, Schwartz, & Pardoll, 1989; Cooper, 2009; Andersen et al., 2000; MacMicking, Taylor, & McKinney, 2003). The immune repertoire is characterized by a complex and dynamic organization, a highly organized, dynamic and coherent structure to assist in the understanding of the generation and selection of immune TCRs. Thus, fully measuring the diversity of the T cell repertoire, which determines the flexibility and specificity in the cellular immune response, could provide new insights into the underlying disease process (Burgos, 1996; Liu et al., 2017). In 1996, Li, et al., investigated disease‐specific change in gamma‐delta T cell repertoire of pulmonary tuberculosis patients by flow cytometric analysis of blood and bronchoalveolar lavage gamma‐delta T cells (Li et al., 1996). They demonstrated the hypothesis that gamma‐delta T cells play a role in the protective immune response to Mtb infection (Li et al., 1996). In 2018, Chaofei Cheng, et al., found that the CDR3δ tended to be more polyclonal and CDR3γ tended to be longer in TB patients; the γδ T cells expressing CDR3 sequences using a Vγ9‐JγP rearrangement expanded significantly during Mtb infection by NGS study of repertoire (Cheng et al., 2018).

However, for further understanding of immune reaction, repertoire diversity and stability within tuberculosis patients and controls still needs more comprehensive studies. Here, we present a study of enormous characterization data of tuberculosis patients and comparable controls. HEC number, HEC ratio, Shannon entropy and Gini coefficient were applied to evaluate the general characteristics of the repertoires. In both patients and normal controls, the HEC number and HEC ratio showed higher frequency in tissue samples than in CD8 or CD4 samples. Besides, HEC number and HEC ratio showed higher frequency in CD8 than CD4 samples in both patients and normal controls. This suggested that a more centralized and stronger immune reaction in tissue samples than in the CD4^+^ or CD8^+^ cell samples provides potential evidence for further elucidation for the understanding of the mechanism in depth. The Shannon entropy which was previously used as an economy parameter, was introduced in this study to illustrate the multiplex of the immune system. According to the criteria, we found that the tissue group's repertoire showed lowest complexity in patients which also suggests the strong immune reaction in tuberculosis tissue than other sample type groups.

The length distribution of CDR3 in each sample was fitted to Gaussian distribution, which provides an evenly distributed data set. Consistent with the previous study, we found that there were significant differences between patients and control groups. Since, the CDR3 sequence which was commonly expressed in patient samples could provide a solid clue for disease‐specific immune reaction research, we evaluated all amino acids shared in all samples. The CD4, CD8 groups showed similar sharing of sequences which were quite different to that of tissue groups. In the analysis of TRBV and TRBJ gene usage, we found significant difference between CD4, CD8 and tissue groups of patients. These differently expressed genes showed a disease‐specific gene expression profile which provides further information for tuberculosis and control study. To find further disease‐specific CDR3 sequences, we also investigated the TRBV/TRBJ combination in patients and controls. Within same group analysis, we found highly expressed combination sequences in each certain group. And we also found differential expression level of recombination sequences in the comparison between patients’ samples and normal control samples, which revealed the important function of TRBV/TRBJ combination in the immune of tuberculosis and provide presupposition for further study of diagnosis or treatment application.

In conclusion, our study first elucidated the immune repertoire characteristics of tuberculosis patients using NGS based methods. We found that the CDR3 sequences were extremely highly expressed in tuberculosis patients’ tissue samples than other type of samples, which suggested a specific and strong immune reaction during the development of tuberculosis. We then analyzed the CDR3 sequence sharing in all samples and each group. Later, we elucidated the TRBV, TRBJ usage and TRBV/TRBJ combination in all samples. This study provides a whole spectrum and profile of tuberculosis patients and studied the specific recombination CRD3 sequences which differ between the patients and controls. Although the sample number is relatively small, we still provide a useful resource of further study on the diagnosis, prognosis and prevention of tuberculosis by understanding candidates’ immunology repertoire features.

## CONFLICT OF INTEREST

The authors have no conflict of interests to declare regarding this manuscript.

## AUTHOR'S CONTRIBUTIONS

YF, BL, SL and YD designed the study and drafted the manuscript. YL, MW, YY, LX, ShL and QH acquired and interpreted the data. SL, YF and YD revised the manuscript for important content. All authors read and approved the final manuscript.

## ETHICS APPROVAL AND CONSENT TO PARTICIPATE

All patients gave written informed consent and the present study was approved by the Medical Ethics Committee of Shenzhen People's Hospital.

## CONSENT FOR PUBLICATION

Not applicable.

## Data Availability

The datasets used and/or analyzed during the current study are available from the corresponding author on reasonable request.
